# A molecular phylogeny of historical and contemporary specimens of an under‐studied micro‐invertebrate group

**DOI:** 10.1002/ece3.7042

**Published:** 2020-12-09

**Authors:** Russell J. S. Orr, Maja M. Sannum, Sanne Boessenkool, Emanuela Di Martino, Dennis P. Gordon, Hannah L. Mello, Matthias Obst, Mali H. Ramsfjell, Abigail M. Smith, Lee Hsiang Liow

**Affiliations:** ^1^ Natural History Museum University of Oslo Oslo Norway; ^2^ Department of Biosciences Centre for Ecological and Evolutionary Synthesis University of Oslo Oslo Norway; ^3^ National Institute of Water and Atmospheric Research Wellington New Zealand; ^4^ Department of Marine Science University of Otago Dunedin New Zealand; ^5^ Department of Marine Sciences University of Gothenburg Gothenburg Sweden

**Keywords:** cheilostome bryozoans, circularized mitochondria, genome‐skimming, high throughput sequencing, historical DNA, museum collections

## Abstract

Resolution of relationships at lower taxonomic levels is crucial for answering many evolutionary questions, and as such, sufficiently varied species representation is vital. This latter goal is not always achievable with relatively fresh samples. To alleviate the difficulties in procuring rarer taxa, we have seen increasing utilization of historical specimens in building molecular phylogenies using high throughput sequencing. This effort, however, has mainly focused on large‐bodied or well‐studied groups, with small‐bodied and under‐studied taxa under‐prioritized. Here, we utilize both historical and contemporary specimens, to increase the resolution of phylogenetic relationships among a group of under‐studied and small‐bodied metazoans, namely, cheilostome bryozoans. In this study, we pioneer the sequencing of air‐dried cheilostomes, utilizing a recently developed library preparation method for low DNA input. We evaluate a de novo mitogenome assembly and two iterative methods, using the sequenced target specimen as a reference for mapping, for our sequences. In doing so, we present mitochondrial and ribosomal RNA sequences of 43 cheilostomes representing 37 species, including 14 from historical samples ranging from 50 to 149 years old. The inferred phylogenetic relationships of these samples, analyzed together with publicly available sequence data, are shown in a statistically well‐supported 65 taxa and 17 genes cheilostome tree, which is also the most broadly sampled and largest to date. The robust phylogenetic placement of historical samples whose contemporary conspecifics and/or congenerics have been sequenced verifies the appropriateness of our workflow and gives confidence in the phylogenetic placement of those historical samples for which there are no close relatives sequenced. The success of our workflow is highlighted by the circularization of a total of 27 mitogenomes, seven from historical cheilostome samples. Our study highlights the potential of utilizing DNA from micro‐invertebrate specimens stored in natural history collections for resolving phylogenetic relationships among species.

## Graphical abstract

Here, we present a workflow that utilizes historical and contemporary specimens, to increase the resolution of phylogenetic relationships among understudied and small‐bodied metazoans, namely, cheilostome bryozoans. In doing so, we present mitochondrial and ribosomal RNA sequences of 43 cheilostomes representing 37 species, including 14 from historical samples ranging from 50 to 149 years old, circularizing a total of 27 mitogenomes, seven from historical cheilostome samples. Our study highlights the potential of utilizing DNA from micro‐invertebrate specimens stored in natural history collections for resolving phylogenetic relationships between species.

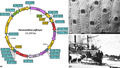

## 1 INTRODUCTION

Robust phylogenetic hypotheses are crucial to understanding many biological processes, ranging from those contributing to population history to those creating macroevolutionary patterns. The development of methods for phylogenetic estimation and high throughput sequencing (HTS) have, in combination, improved our understanding of the relationships among extant organisms. These advances are frequently applied to the estimation of relationships among higher taxa, for example, distantly related animal phyla (Laumer et al., 2019; Simion et al., 2017), families of flowering plants (Léveillé‐Bourret et al., 2017), and orders of birds (Jarvis et al., 2014). While these deeper branches have received significant attention, those at the leaves (e.g., genera and species) often remain largely unresolved, especially for taxa that are under‐studied. For the resolution of relationships at lower taxonomic levels, crucial as a backbone for answering many evolutionary questions, a rich and broad species representation is vital.

Traditionally, researchers have sequenced relatively freshly collected specimens. However, there is an increasing utilization of historical specimens for molecular phylogenetic reconstruction in the absence of fresh tissue (e.g., Jarvis et al., 2014). Historical specimens from museum or other institutional collections are valuable sources of information representing organisms that may be difficult or impossible to sample in contemporary populations (Holmes et al., 2016). Most studies that use historical specimens for phylogenetic reconstruction tend to focus on larger‐bodied species that are recently extinct (Anmarkrud & Lifjeld, 2017; Bunce et al., 2009; Mitchell et al., 2014; Sharko et al., 2019), well‐studied groups (Billerman & Walsh, 2019; Jarvis et al., 2014) and organisms of economic importance (Bonanomi et al., 2014; Larson et al., 2007; Larsson et al., 2019). Only recently have we begun to see an increase in such reports on invertebrate and other small‐bodied taxa (e.g., Cruaud et al., 2019; Der Sarkissian et al., 2017; Kistenich et al., 2019; Sproul & Maddison, 2017). When employing HTS in such historical studies, nucleotide reads are typically mapped to a closely related reference genome, or one from the same species (Sharko et al., 2019). Alternatively, complete sequences of target regions from related species and/or genera (Anmarkrud & Lifjeld, 2017; Billerman & Walsh, 2019) are used to design baits or probes (Derkarabetian et al., 2019; Ruane & Austin, 2017). While these approaches are excellent for groups whose sequences are relatively well‐understood, they are unfeasible for clades with low inter‐genus sequence conservation, or for those lacking sequence data from closely related reference organisms, such as those we will present here.

Here, we use historical and contemporary material from lesser‐studied, small‐bodied organisms for the purpose of reconstructing robustly supported phylogenetic relationships using molecular data. Our target organisms are cheilostomes, the most species‐rich order of the phylum Bryozoa, with ca. 6,500 described extant species, representing about 80% of the living species diversity of the phylum (Bock & Gordon, 2013). Cheilostomes are lightly to heavily calcified, sessile, colonial metazoans common in benthic marine habitats. Most species are encrusting, while fewer are erect, and most are small (colony size c. 1 cm^2^, module size c. 500 μm × 200 μm), and live on hard substrates that may be overgrown by other fouling organisms (including other bryozoans, hydroids, foraminifera, and tube worms).

Systematic relationships among cheilostome bryozoans remain largely based on morphological characters (Bock & Gordon, 2013) with molecular phylogenies being restricted to recently collected, ethanol‐preserved samples where genetic data were obtained using PCR‐based methods (Fuchs et al., 2009; Knight et al., 2011; Orr, Waeschenbach, et al., 2019; Waeschenbach et al., 2012), or more recently, by a HTS genome‐skimming approach (Orr, Haugen, et al., 2019). While these studies have improved our understanding of the phylogenetic relationships among cheilostomes, many key taxa, potentially filling important phylogenetic positions, are hard to procure and remain unfeatured in sequencing projects. Contributing to the advancement of historical DNA methods (Billerman & Walsh, 2019; Knapp & Hofreiter, 2010), we pioneer the sequencing of air‐dried bryozoan specimens (i.e., never preserved in ethanol) that have been stored up to 150 years since collection. To do so, we employ a recently developed DNA library preparation method (SMARTer ThruPLEX, Takara) to amplify and sequence historical samples with low DNA concentrations. We bypass the need for primers/probes, a clear advantage for cheilostomes which are known to have low inter‐genus sequence conservation (Orr, Haugen, et al., 2019; Orr, Waeschenbach, et al., 2019; Waeschenbach et al., 2012). We use the de novo mitogenome assembly from the target colony itself as a reference for iterative mapping so as to avoid difficult assumptions in reference selection especially in the cases where no conspecifics have been sequenced before. In doing so, we generate mitochondrial and ribosomal RNA sequences of 43 cheilostome colonies representing 37 species and 30 genera, with 14 of these from historical samples ranging from 50 to 149 years old (Table S1). As a derivative of the demonstration of our workflow, we present a well‐supported cheilostome tree using 65 ingroup taxa and 17 genes, the largest and taxonomically most broadly sampled cheilostome phylogeny to date. Finally, the success of the methodology we employed is highlighted by the circularization of 27 cheilostome mitochondrial genomes, seven of which were from historical samples. This approach emphasizes the potential for analyzing DNA from micro‐invertebrate samples stored in natural history collections, especially for phylogenetic reconstruction of many hitherto inaccessible cheilostome genomes.

## 2 MATERIALS AND METHODS

Twenty‐one dried historical cheilostome samples and 29 recently collected samples, of which 26 were ethanol‐preserved and three dried, were targeted (Table 1 and Table S1). We selected the historical samples to represent a spread of collection dates while including those that are already phylogenetically resolved (i.e., their phylogenetic placements are known and/or we have available contemporary samples of their conspecifics or congenerics) and those that are currently enigmatic. Recently, collected samples were selected for their potential for phylogenetic verification of the historical specimens. Each colony was subsampled for DNA isolation and scanning electron microscopy (*SEM*), using a Hitachi TM4040PLus. For microscopy, we bleached subsamples in diluted household bleach for a few hours to overnight, removing soft tissues in order to document skeletal morphology. SEMs of dried samples were taken both pre‐ and post‐bleaching. All physical vouchers necessary for taxonomic verification are stored at the Natural History Museum of Oslo, University of Oslo, and SEMs are available in the Online Supporting Information (SI) as *SEM* cards familiar to bryozoologists. Additional sample metadata are reported in Table S1.

**Table 1:** Samples generated and analyzed in this study

Genus	Species	BLEED	Year	Country	rRNA	Mt	Mt size (bp)	Accession
*Antarctothoa*	*delta*	703	2018	NZ	2	12	14,114	MT311319, MT311464, MT293076
*Arachnopusia*	*unicornis*	183	2009	NZ	2	15	NO	MT311320, MT311465, MT293114, MT293115
*Arachnopusia*	*unicornis*	221	2011	NZ	2	15	NO	MT311321, MT311466, MT293085
*Bicellariella*	*ciliata*	560	2018	SWE	2	15	19,704	MT311322, MT311467, MT293086
*Bugula*	*neritina*	1,200	1960*	AUS	2	15	15,414	MT311323, MT311468, MT293098
*Caberea*	*angusta*	160	2009	NZ	2	13	NO	MT311324, MT311469, MT293118
*Caberea*	*ellisii*	1,190	<1970*	NOR	0	14	17,589	MT311325, MT311470, MT293122
*Calpensia*	*nobilis*	1,184	<1970*	IT	2	14	14,049	MT311326, MT311471, MT293091
*Celleporina*	*sinuata*	328	2015	NZ	1	14	15,651	MT311327, MT293103
*Cornuticella*	*trapezoidea*	1,687	2018	NZ	2	14	14,590	MT311328, MT311472, MT293078
*Cradoscrupocellaria*	*reptans*	1,192	<1970*	NO	1	15	NO	MT311350, MT293075
*Electra*	*pilosa*	819	2018	NO	2	14	13,970	MT311330, MT311474, MT293123
*Electra*	*pilosa*	1,172	2018	NO	2	13	13,965	MT311329, MT311473, MT293093
*Electra*	*pilosa*	1,178	<1970*	‐	0	4	NO	MT293096
*Electra*	*scuticifera*	98	2015	NZ	2	12	13,804	MT311331, MT311475, MT293082
*Emma*	*tricellata*	196	2015	NZ	2	14	16,275	MT311332, MT311476, MT293080
*Flustra*	*foliacea*	568	2018	SWE	2	15	16,802	MT311333, MT311477, MT293116
*Hincksina*	sp. nov.	61	2009	NZ	2	14	15,726	MT311334, MT311478, MT293104
*Hippothoa*	sp.	1,187	1875*	UK	0	15	NO	MT311335, MT311479, MT293117
*Membranipora*	*membranacea*	816	2018	NO	2	15	14,739	MT311337, MT311481, MT293110
*Membranipora*	*membranacea*	1,163	2018	SWE	2	15	14,736	MT311336, MT311480, MT293090
*Myriapora*	*truncata*	1,197	1871*	IT	2	15	NO	MT311338, MT311482, MT293097
*Omalosecosa*	*ramulosa*	1,177	<1970*	NO	1	8	NO	MT311339, MT293108, MT293112
*Oshurkovia*	*littoralis*	1,194	<1970*	UK	2	13	13,973	MT311340, MT311483, MT293087
*Parasmittina*	*aotea*	86	2010	NZ	2	13	14,232	MT311341, MT311484, MT293094
*Parasmittina*	*jeffreysi*	1,202	1901*	Arctic	2	15	14,260	MT311342, MT311485, MT293102
*Parasmittina*	*solenosmilioides*	1,267	2015	NZ	2	15	17,725	MT311343, MT311486, MT293113
*Patsyella*	*acanthodes*	131	2011	NZ	2	14	NO	MT311344, MT311487, MT293109
*Porella*	*compressa*	1,188	<1970*	NO	1	5	NO	MT311488, MT293089, MT293083, MT293105
*Porella*	*concinna*	579	2018	SWE	2	13	14,277	MT311346, MT311490, MT293095
*Porella*	*concinna*	1,169	2018	SWE	2	14	14,278	MT311345, MT311489, MT293084
*Pterocella*	*scutella*	104	2015	NZ	2	13	14,025	MT311347, MT311491, MT293081
*Rhabdozoum*	*wilsoni*	695	2018	NZ	0	15	NO	MT293111
*Rhynchozoon*	*angulatum*	694	2018	NZ	2	13	14,372	MT311348, MT311492, MT293088
*Rhynchozoon*	*zealandicum*	127	2015	NZ	2	12	14,092	MT311349, MT311493, MT293099
*Securiflustra*	*securifrons*	551	2018	SWE	2	8	NO	MT311352, MT311495, MT293077
*Securiflustra*	*securifrons*	1,179	<1970*	NO	2	15	17,457	MT311351, MT311494, MT293106
*Steginoporella*	*perplexa*	100	2011	NZ	2	12	13,700	MT311353, MT311496, MT293100
*Stephanollona*	aff. *scintillans*	679	2018	NZ	1	12	NO	MT311354, MT293120
*Stephanollona*	*scintillans*	170	2009	NZ	2	8	NO	MT311355, MT311497, MT293101, MT293107, MT293119
*Terminocella*	n. sp.	194	2010	NZ	2	15	NO	MT311356, MT311498, MT293121
*Tessaradoma*	*boreale*	1,183	<1970*	NO	2	13	14,211	MT311357, MT311499, MT293079
*Turbicellepora*	*smitti*	1,182	<1970*	NO	0	14	NO	MT293092

Note

Genus and species names are given, followed BLEED (numbers) which are numerical tags for the specimens. BLEED stands for Bryozoan Lab for Ecology, Evolution and Development. Year refers to collection year. An * succeeding the year of collection indicates an air‐dried historical sample, defined here as 50 years or older. Multiple historical samples have “<1970” indicated because the collection year was unstated. However, taxonomic identifications were made in 1970 for these specimens, implying that they must have been collected then, or earlier. Abbreviations for countries (approximate locations where the sample was collected) are as follows: NZ = New Zealand, SWE = Sweden, AUS = Australia, NOR = Norway, IT = Italy and UK = United Kingdom. The columns “rRNA” and “Mt” represent the number of rRNA and mitochondrial genes annotated and used in phylogenetic inference, with a maximum of 2 and 15, respectively. The size of the mitogenome, in base pairs (bp), is only shown if closed/circularized, otherwise the cell is labeled as “NO.” For an expanded overview of metadata, see Table S1.

### 2.1 DNA isolation, sequencing, assembly, and annotation

DNA from the 21 historical specimens was isolated in a laboratory designed for handling samples with low DNA concentrations (sensi‐lab, NHM, Oslo). DNA extractions were performed inside a hood equipped with UV lights and all equipment was bleached and UV‐sterilized prior to use. Samples were vortexed twice in nuclease‐free H_2_O, air‐dried, then subject to UV for 10 min to minimize possible surface contaminants. These treated samples were subsequently crushed with a stainless‐steel mortar and pestle (Gondek et al., 2018). To minimize the risk of sample cross‐contamination, each historical sample was processed separately prior to DNA isolation. The 29 contemporary samples were isolated in a standard laboratory without the aforementioned surface decontamination and crushing steps.

Genomic DNA for all 50 specimens was isolated using the DNeasy Blood and Tissue kit (QIAGEN). Colonies were homogenized in lysis buffer, using a pestle, in the presence of proteinase‐K prior to a 16‐hr 56°C incubation period. An on‐column RNase A step was additionally performed to obtain RNA‐free genomic DNA. DNA was eluted in pre‐heated 60°C Tris‐Cl buffer (10 mM) and incubated at 37°C for 10 min. Recovered DNA was quantified prior to library preparation using a Qubit 2.0 fluorometer (ThermoFisher).

Sequencing libraries were prepared with the standard KAPA HyperPrep kit (Roche) by the Norwegian Sequencing Centre (NSC) for DNA samples >15 ng. For those with <15 ng DNA, the SMARTer^®^ ThruPLEX^®^ DNA‐Seq Kit (Takara Bio Inc) was used in the sensi‐lab at NHM Oslo (Table S1). The contemporary DNA samples were fragmented to a 350 bp insert size, while no such step was performed on the historical samples with already short DNA length, giving a variable insert size (Table S2). Unique dual indexes (UDIs) were used once per library and any unligated adapter removed. Libraries constructed from the KAPA and SMART preparation methods were each pooled, then separately loaded on two independent flow cells prior to multiplex sequencing with independent runs. Genomic DNA up to 150 bp in read length was pair‐end (PE) sequenced on an Illumina HiSeq4000 at the NSC. A blank control, taken during the DNA extraction of the historical samples, was also sequenced (library preparation: SMARTer^®^ ThruPLEX^®^ DNA‐Seq Kit). Illumina HiSeq reads were quality checked using FastQC v.0.11.8 (Andrews, 2010), then quality‐ and adapter‐trimmed using TrimGalore v0.4.4 with a Phred score cutoff of 30 (Krueger, 2015). Trimmed reads were de novo assembled with SPAdes 3.11.1 (Bankevich et al., 2012) using k‐mers of 21,33, 55, 77, 99, and 127. The mitogenome and rRNA operon of each sample were identified separately with blastn (Altschul et al., 1990) using blast + against a database constructed from broadly sampled cheilostome sequences already deposited in NCBI (Table S7). An E‐value of 1.00e‐185 and maximum target sequence of 1 were used to filter any blast hits of non‐ cheilostome origin. We use three current assembly methods and compare their potential for the recovery of mitogenome sequences from our data and subsequent phylogenetic inference of our historical samples. For each historical sample, the SPAdes de novo assembled mitogenome sequence was used as the reference (seed) input for its own iterative mapped assembly using a relatively new but already widely‐used method, GetOrganelle (Jin et al., 2020) and another commonly used method, NOVOplasty 3.7 (Dierckxsens et al., 2016), both under default settings. In addition, a mitogenome sequence from a taxon phylogenetically related to the historical sample in question was also provided for a second iterative assembly (Figure S1). Both iterative methods of GetOrganelle and NOVOplasty work by mapping sequencing reads to a reference, or seed, before de novo extension of contig ends. Circularization (or closure) of each mitochondrial genome was confirmed using blast2 (Altschul et al., 1990), with the same sequence used as query and reference to validate overlap of contig ends. The overlapping mitochondrial sequence region was subsequently trimmed manually.

### 2.2 Annotation and alignments

**Figure 1:**
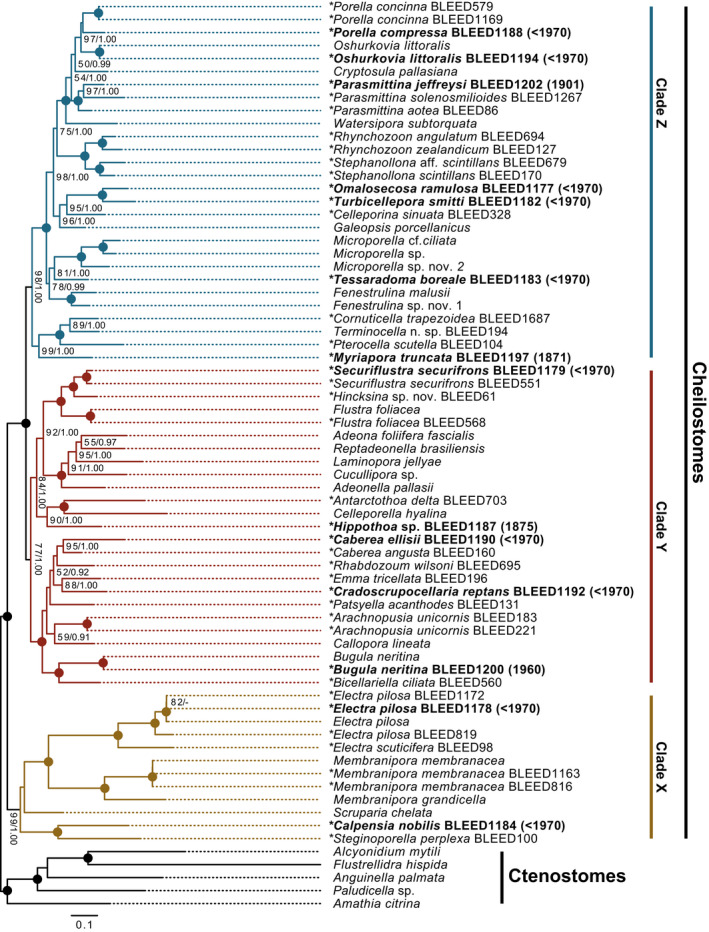
The inferred phylogeny of cheilostomes based on 17 genes from historical and recently sampled material. Maximum likelihood topology of 65 cheilostome ingroup taxa and 5 ctenostome outgroup taxa with 8,324 nucleotide and amino acid characters inferred using RAxML (100 heuristic searches and bootstrap of 500 pseudoreplicates). The numbers on the internal nodes are ML bootstrap values (BS from RAxML) followed by posterior probabilities (PP from MrBayes). Circles indicate 100 BP and 1.00 PP. Only BS > 50 and PP > 0.95 are shown, dash indicates values below this. * indicates taxa generated in this study. Bold font indicates historical samples with sampling year in brackets. Clade X (blue), Clade Y (red), and Clade X (brown) discussed in the supporting text are highlighted. See Table S3 for genes available and Table S4 for % missing characters for each taxon

Mitogenomes from the three separate assembly methods, for each of the samples, were annotated with Mitos2 using a metazoan reference (RefSeq 89) and the invertebrate genetic code (Bernt et al., 2013) to identify two rRNA genes (rrnL and rrnS) and 13 protein coding genes (*atp6, atp8, cox1, cox2, cox3, cob, nad1, nad2, nad3, nad4, nad4l, nad5,* and *nad6*). In addition, two rRNA operon genes (ssu (18s) and lsu (28s)) were identified and annotated using RNAmmer (Lagesen et al., 2007). Further, mitogenes and rRNA operons from 27 bryozoan taxa (Table S6), obtained from NCBI, selected for their potential to verify the success of our workflow for our historical specimens, were aligned with our samples to compile a broader taxon sample representing a cheilostome ingroup and ctenostome outgroup. A minimum gene number of four per taxon, to avoid possible negative effects of missing data on phylogenetic inference (Philippe et al., 2004; Wiens & Morrill, 2011), was set for this study. The number of genes included for each taxon is shown in Table 1, while sequence length per gene and % missing characters (nucleotide or amino acid) per taxon are shown in Table S4. MAFFT (Katoh & Standley, 2013) was used for alignment with default parameters: for the four rRNA genes (nucleotide) the Q‐INS‐i model, considering secondary RNA structure, was utilized; for the 13 protein coding genes, in amino acid format, the G‐INS‐I model was used. The 17 separate alignments were edited manually using Mesquite v3.1 to remove any uncertain characters (Maddison & Maddison, 2017). Ambiguously aligned characters were removed from each alignment using Gblocks (Talavera & Castresana, 2007) with least stringent parameters. The single‐gene alignments were concatenated to a supermatrix using the catfasta2phyml perl script (Nylander, 2010). An initial mitochondrial supermatrix, consisting of up to five assemblies per sample (Figure S1), was used to compare and evaluate differences among the three assembly methods via a phylogenetic analysis (see next section). Using only a single assembly method per taxon from this initial supermatrix of historical samples, we then created a downstream final supermatrix. Each sample (i.e., those presented in Table 1) was represented only once in the final supermatrix to which the two rRNA operon genes from the SPAdes de novo assembly were added (Figure 1). The alignments (both masked and unmasked) are available through Dryad (https://doi.org/10.5061/dryad.bk3j9kd7m).

### 2.3 Phylogenetic reconstruction

Maximum likelihood (ML) phylogenetic analyses were carried out for each single‐gene alignment using the “AUTO” parameter in RAxML v8.0.26 (Stamatakis, 2006) to establish the evolutionary model with the best fit. The general time reversible (GTR + G) was the preferred model for the four rRNA genes (18s, 28s, rrnS, and rrnL), and MtZoa + G for all 13 protein coding genes. The concatenated datasets, divided into rRNA and protein gene partitions, each with its own separate gamma distribution were analyzed using RAxML. The topology with the highest likelihood score of 100 heuristic searches was chosen. Bootstrap values were calculated from 500 pseudoreplicates.

Bayesian inference (BI) was performed using a modified version of MrBayes incorporating the MtZoa evolutionary model (Huelsenbeck & Ronquist, 2001; Tanabe, 2016) for the samples represented in the final supermatrix (Figure 1). The dataset was executed, as before, with rRNA and protein gene partitions under their separate gamma distributions. Two independent runs, each with three heated and one cold Markov Chain Monte Carlo (MCMC) chain, were initiated from a random starting tree. The MCMC chains were run for 20,000,000 generations with trees sampled every 1,000th generation. The posterior probabilities and mean marginal likelihood values of the trees were calculated after the burnin phase (5,000,000 generations). The average standard deviation of split frequencies between the two runs was <0.01, indicating convergence of the MCMC chains.

## 3 RESULTS

We successfully sequenced and assembled 43 cheilostome colonies representing 37 species from 30 genera (Table 1). The correspondence between the phylogenetic placement of each colony and its identity based on morphology was confirmed using the *SEM* vouchers (SI). This verification is important for checking if sequences from a sample, be they contemporary or historical, are contaminated with non‐target cheilostomes due to the benthic, sessile, largely encrusting life‐habit. Fourteen of these colonies came from historical samples, each representing a separate genus. The oldest known historical sample we assembled was of *Myriapora truncata* (BLEED 1,197) from the year 1871. The 14 successfully assembled historical cheilostome samples had a total DNA input range of 1.3–304 ng for Illumina library preparation (Table S2). Of the 21 sequenced historical samples, one failed to provide any sequence read data. Of the 20 assembled historical samples, one was removed from the dataset based on our minimum gene number criterion for phylogenetic inference (Table S3). For four of the 20 assembled samples, no cheilostome mitogenome or rRNA operon genes were identified with the utilized blastn parameters (see methods and Table S3). And for one sample, the assembly provided a limited contig number with no cheilostome target identifiable. The negative control provided 3.6 million reads that upon assembly gave 50 contigs >500 bp, with a 1,561 bp maximum. All assembled contigs from the negative control were attributed to contamination from *Canis familiaris* and *Homo sapiens*. No contigs from the negative control were attributed to cheilostome contaminants.

The three assembly methods (GetOrganelle, NOVOplasty, and SPAdes) yielded assemblies that resulted in a high level of phylogenetic congruence with one another, where the nodes subtending each sample formed fully supported monophylies (Figure S1). This result is independent of the initial seed sequence (i.e., the sample itself or a conspecific or another closely related taxon) used for GetOrganelle and NOVOplasty, the two iterative methods. The only discrepancy was that of *Porella compressa* (BLEED 1,188) assembled using NOVOplasty with *P. concinna* (BLEED 579) as seed, where BLEED 1,188 grouped with the BLEED 579, rather than itself. Note that the NOVOplasty assembly of BLEED 1,188 using BLEED 579 was not used in downstream analyses that resulted in Figure 1, as only the single most complete assembly for each sample was used for the final phylogenetic reconstruction. The genes that are recovered using different assembly methods varied for multiple specimens. The de novo method using SPAdes recovered the most mitochondrial genes per sample on average (Figure S1 and Table S5). This was followed by the iterative method of NOVOplasty, with its results independent of seed input. Lastly, GetOrganelle consistently recovered the fewest mitochondrial genes per sample. Additionally, GetOrganelle failed to assemble in approximately 40% of cases.

Given the phylogenetic congruence of the assembly methods (Figure S1), we concatenated genes from these separate assemblies to form a final supermatrix with each sample represented only once (see methods and Table 1) to infer the phylogeny presented in Figure 1. Specifically, the input for each specimen to this supermatrix utilized the assembly method that gave the highest number of annotated genes. With an equal number of recovered genes between assemblies for a given sample, prioritization was as follows (Figure S1; star indicates assembly utilized in Figure 1): the de novo assembly of SPAdes (9 taxa), before that of NOVOplasty utilizing a SPAdes seed (3 taxa), and lastly NOVOplasty using a seed from a close relative (2 taxa). No assembly from GetOrganelle was used in the final supermatrix. Sequence length, and % missing characters, for each taxon and gene utilized in the main phylogeny (Figure 1) are presented as supporting data (Table S4). The final inferred supermatrix constituted 70 taxa, and 8,324 nucleotide and amino acid sites with a relatively low 27.6% total missing characters.

The main phylogeny (Figure 1) is robustly resolved with most branches and relationships receiving either high (>90 bootstrap (BS)/ 0.99 Posterior Probability (PP)) or full support (100 BS/ 1.00 PP). The tree topology and their implications for our understanding of cheilostome evolutionary relationships are presented in detail in the supporting information (SI). Briefly, focusing specifically on the historical samples generated in this study we observe that *Bugula neritina* (BLEED 1,200), *Electra pilosa* (BLEED 1,178), *Oshurkovia littoralis* (BLEED 1,194), and *Securiflustra securifrons* (BLEED 1,179) all form fully supported monophyletic intraspecies relationships (Figure 1), verified by their placement with contemporary specimens of the same species. *Caberea ellisii* (BLEED 1,190) and *Parasmittina jeffreysi* (BLEED 1,202) form highly (95 BS/ 1.00 PP) and fully supported monophylies, respectively, within their respective genera, and again verified using recently collected samples (*C. angusta* BLEED 160, *P. solenosmilioides* BLEED 1,267 and *P. aotea* BLEED 86). Further, *Porella compressa* (BLEED 1,188) places within a genus monophyly (to the contemporary samples *P. concinna* BLEED 579 and BLEED 1,169), although statistical support is weak. *Hippothoa* sp. (BLEED 1,187), *Omalosecosa ramulosa* (BLEED 1,177) *Turbicellepora smitti* (BLEED1182), and *Cradoscrupocellaria reptans* (BLEED 1,192) are all highly supported within their respective corresponding families (see SI), with their placements corroborated from the inclusion of the contemporary samples *Antarctothoa delta* BLEED 703, *Celleporina sinuata* BLEED 328, and *Emma tricellata* BLEED 196. The three remaining taxa, *Calpensia nobilis* (BLEED 1,184), *Myriapora truncata* (BLEED 1,197), and *Tessaradoma boreale* (BLEED 1,183) all lack the inclusion of a close taxonomic relative in our phylogenetic inference. However, for *M. truncata* (BLEED1197) a phylogeny of *cox1* (Figure S2) demonstrates that our historical sample forms a fully supported monophyly with that of a *M. truncata cox1* sequence obtained from NCBI (ATX63952).

**Figure 2:**
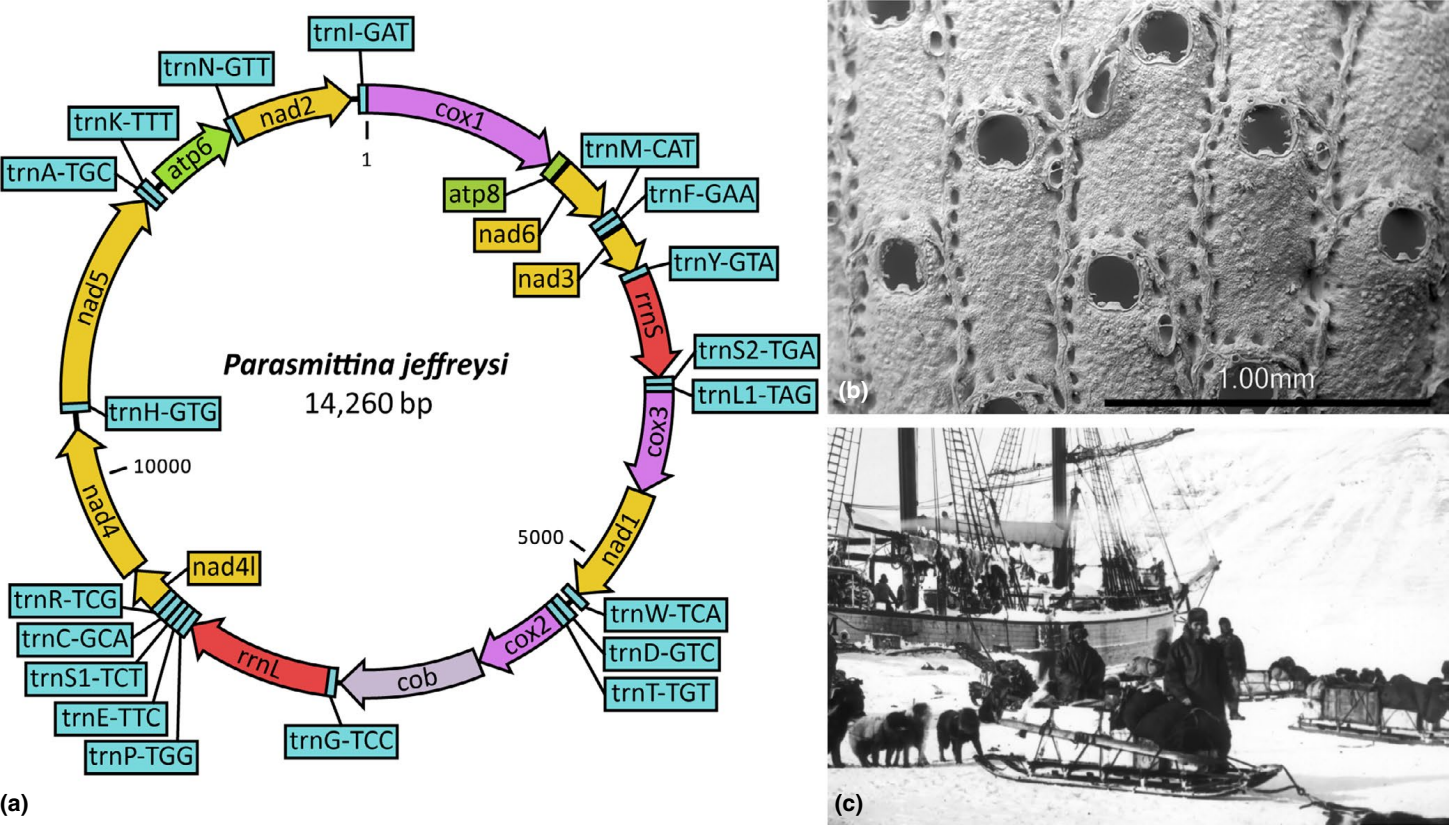
The circularized mitochondrial genome of *Parasmittina jeffreysi* (BLEED 1,202). The figure shows (a) the circularized 14,260 bp mitogenome of *P. jeffreysi* (BLEED1202, collected in 1901) with gene order and direction of transcription (indicated by block arrows). *SEM* (b) of the same sample, and a photograph (c) from the 1901 Norwegian FRAM II expedition where this sample was collected. The FRAM II picture is used with permission of the Norwegian Polar Institute

In total, mitochondrial genomes of 27 of 43 samples generated from this study were circularized with a size range of 13,700–19,704 bp (Table 1). Of these, seven were from the 14 historical samples, with the oldest being that of *Parasmittina jeffreysi* (BLEED 1,202, 14,260 bp, collected in 1901; Figure 2).

## 4 DISCUSSION

Continued explorative expeditions and the discovery of yet unknown organisms will provide optimal organic material for DNA sequencing, and subsequent analyses of extant species. However, natural history collections are unique sources that can provide historical material for organisms that may be difficult or impossible to sample from contemporary populations. Experimentation with laboratory techniques and bioinformatic tools of such historical samples can expand the information space for our general understanding of the biology, including the genetics and phylogenetics, of both well‐ and under‐studied species. In this study, we expanded on our knowledge of a small‐bodied and under‐studied phylum, by applying HTS and a combination of de novo and iterative assembly methods. We demonstrated that historical samples can be used to boost inference of the phylogenetic relationships among a micro‐invertebrate group that is challenging to work with, namely bryozoans.

### 4.1 Overcoming challenges of sequencing under‐studied groups

While the cheilostome molecular tree is very far from complete (Fuchs et al., 2009; Knight et al., 2011; Orr, Haugen, et al., 2019; Orr, Waeschenbach, et al., 2019; Waeschenbach et al., 2012), we have contributed to this community endeavor by sequencing a number of species that have never been sequenced before and by demonstrating that it is possible, without much extra effort, to extract sequence data of many genes from old, air‐dried samples. We utilized de novo and iterative methods to assemble our cheilostome mitochondrial sequence data to overcome the challenges of reference‐based assemblies, due to the high degree of observed sequence variability among cheilostomes. This is especially important because systematic relationships remain largely based on morphological characters for cheilostomes (Bock & Gordon, 2013) which we know can sometimes be misleading (Orr, Haugen, et al., 2019; Orr, Waeschenbach, et al., 2019); hence, reference choice is fraught with difficulties. By using de novo assembled sequences from the target colony itself as a reference for iterative mapping, we circumvent the difficulties of reference choice. To validate our approach, we also explored the robustness of the phylogenetic placement by using both nominal conspecifics and congenerics as references where available (Figure S1). Encouragingly, we find that historical samples can be used as their own reference for iterative mapping assemblies, at least for the samples we studied. Phylogenetic comparison of these assemblies consistently demonstrated fully supported monophylies, independent of method and the supplied reference. While SPAdes (de novo), in general, recovered the most complete assemblies and mitochondrial genes, the output of NOVOplasty (iterative) was almost comparable, and better in some cases. We propose that the iterative method employed in NOVOplasty could supplement that of the de novo method, beckoning integration into mitogenome assembly pipelines, and removing the necessity for a reference sequence from a closely related species. The latter is especially relevant in under‐studied groups, such as cheilostomes, where it is not always clear how closely related different species are. Conversely, the use of GetOrganelle was superfluous for our dataset, recovering the fewest genes per sample, and having the highest failure rate. The iterative methods of GetOrganelle and NOVOplasty have previously been compared for chloroplast assemblies (Freudenthal et al., 2019; Oliveira et al., 2020) and combined for plastid genome construction (Ma & Lu, 2019; Tan et al., 2019; Yan et al., 2019). Results from these previous comparative analyses, in contrast to our own, indicate GetOrganelle may be more suited to chloroplast assemblies. Finally, the workflow we describe in the present study proved suitable for circularizing mitochondrial genomes (27 samples, ranging from 13,700 to 19,704 bp; Table 1), including from historical samples >100 years old (Figure 2). While detailed mitogenome comparisons are beyond the scope of this current study, they are now available for further genome evolution research.

### 4.2 Phylogenetic inference, missing data and taxon sampling

The phylogeny presented here is the most broadly taxonomically sampled and resolved cheilostome tree to date. The statistical support of the majority of our branches (Figure 1) indicate that missing data have little impact on the phylogenetic inference for our taxon sample (Wiens & Morrill, 2011). To highlight this point, the five samples with the highest percentage of missing characters in the final inferred supermatrix (*Electra pilosa* BLEED1178, 84% missing; *Fenestrulina malusii*, 76% missing; *Microporella* sp. nov. 2, 74% missing; *Microporella* cf. *ciliata,* 73% missing; and *Porella compressa,* BLEED1188 73% missing) all place robustly, with full support to their respective species or genus (see Table S4 and Figure 1), indicating that the available data are phylogenetically informative. A broad taxon sample combined with a relatively low proportion of total missing characters in the final supermatrix (27.6%, see Table S4) has contributed to the high degree of phylogenetic resolution observed (Wiens & Morrill, 2011).

The recent introduction of HTS and genome‐skimming to bryozoology has brought overdue support to phylogenetic relationships, but this approach is not without its challenges (Orr, Haugen, et al., 2019; Orr, Waeschenbach, et al., 2019). Any given cheilostome bryozoan colony (sample) usually lives in close proximity with other fouling/encrusting organisms, including other bryozoan species, so contamination is always a concern when isolating and amplifying target DNA, independent of sample age. We alleviated this challenge by using phenotypic information from our physical and *SEM* vouchers (SI). Further, we employ a bioinformatic pipeline with robust filtering steps to eliminate sequences from non‐target contaminants (Orr, Haugen, et al., 2019). Lastly, inclusion of contemporary conspecifics and congenerics in our study confirm phylogenetic placement of the targeted bryozoans (Figure 1). The purpose of demonstrating reliable phylogenetic placement of historical samples with respect to available conspecifics, congenerics, or other close relatives is to establish methodological confidence for cases in which samples lacked such controls. For instance, additional specimens of *Calpensia, Myriapora,* and *Tessaradoma,* contemporary or historical, were unavailable for this study, and hence the phylogenetic placement of BLEED1184, BLEED1197, and BLEED1183 cannot be verified with a close relative. However, other information may help to verify specific samples. Firstly, and discussed in detail in the accompanying supporting text, *Calpensia* (BLEED1184) shares important morphological traits with *Steginoporella* and its relatives (see SEMs in SI) even though it currently nominally belongs to the phylogenetically more derived, but one‐size‐fits all family Microporidae (Gray, 1848; Jullien, 1888). Secondly, the *Myriapora cox1* gene has previously been amplified utilizing barcoding primers (Cahill et al., 2017). We can therefore confirm the placement of *Myriapora* (BLEED1197) in the main phylogeny (Figure 1) based on 17 genes, by showing its *cox1* affinity to that of *Myriapora cox1* from NCBI (Figure S2). Lastly, the position of *Tessaradoma* (BLEED1183), whose taxonomic identity is confirmed by its *SEM* voucher, as sister to *Microporella* remains a working hypothesis until increased taxon sampling can either confirm or reject this position. We emphasize that nothing can substitute for the continued accumulation of broadly taxonomically sampled sequence and vouchered morphological data for aiding phylogenetic understanding. With the exception of *Tessaradoma* (see above), the confirmed phylogenetic placement of all of our 14 historical samples supports that any effect of possible non‐target contaminant in the tree (Figure 1) can be excluded. In our study, the negative control provided only a limited read number that upon assembly gave few and short contigs attributed to mammal DNA, which were filtered out during the assembly pipeline. Importantly, no cheilostome DNA was observed in the negative control.

### 4.3 The importance of natural history collections

Natural history collections are treasure‐troves of past information that can potentially shed light on many questions that we may not even have yet thought of, with tools that are continuously being developed. We have capitalized on historical samples stored at the Museum of Natural History at the University of Oslo, accumulated during periods where future sequencing technologies or phylogenetic estimation techniques belonged to the realm of science fiction (Short et al., 2018; Yeates et al., 2016). Many more such collections exist throughout the world and our relatively simple workflow widens the possibilities of extracting useful sequence information from many more under‐studied phyla and small‐bodied invertebrates, beyond cheilostome bryozoans. This work illustrates the importance of natural history collections, and the need to store and maintain these for future generations and technological advances.

## CONFLICT OF INTEREST

None declared.

## AUTHOR CONTRIBUTION

**Russell Orr:** Conceptualization (lead); Data curation (equal); Formal analysis (lead); Investigation (equal); Methodology (lead); Project administration (lead); Resources (equal); Software (equal); Supervision (lead); Validation (lead); Visualization (equal); Writing‐original draft (equal); Writing‐review & editing (equal). **Maja Sannum:** Formal analysis (equal); Investigation (equal); Methodology (equal); Project administration (equal); Validation (equal); Writing‐original draft (equal). **Sanne Boessenkool:** Conceptualization (equal); Investigation (supporting); Methodology (supporting); Supervision (supporting); Validation (supporting); Writing‐original draft (supporting); Writing‐review & editing (equal). **Emanuela Di Martino:** Formal analysis (supporting); Investigation (supporting); Methodology (supporting); Validation (supporting); Visualization (supporting); Writing‐review & editing (equal). **Dennis Gordon:** Validation (supporting); Visualization (supporting); Writing‐original draft (equal); Writing‐review & editing (equal). **Hannah Melo:** Resources (supporting); Writing‐review & editing (equal). **Matthias Obst:** Resources (supporting); Writing‐review & editing (equal). **Mali Ramsfjell:** Validation (equal); Visualization (equal); Writing‐review & editing (equal). **Abigail Smith:** Resources (equal); Writing‐review & editing (equal). **Lee‐Hsiang Liow:** Conceptualization (equal); Formal analysis (equal); Funding acquisition (lead); Investigation (equal); Methodology (equal); Project administration (equal); Supervision (equal); Validation (equal); Visualization (equal); Writing‐original draft (equal); Writing‐review & editing (equal).

## AUTHOR CONTRIBUTIONS

RJSO, SB, and LHL: designing the study; HM, AMS, DPG, MO, RJSO, LHL, and MHR: collecting the material; RJSO and MMS performing the laboratory work and bioinformatics; MHR and EDM: taking the SEMs, DPG, EDM, MO identified the taxa; RJSO and LHL: writing the first draft of the manuscript which all authors commented and agreed on.

## Supporting information

FigS1‐S18

TableS1‐S7

Supplementary Material

Supplementary Material

## Data Availability

Sequences generated and used in this study have been submitted to GenBank and accessions are provided. The alignments (both masked and unmasked) used in the phylogenetic inferences are freely available through Dryad (https://doi.org/10.5061/dryad.bk3j9kd7m). All physical vouchers are stored at the Natural History Museum of Oslo, University of Oslo.
