# Supplementary material for: A molecular phylogeny of historical and contemporary specimens of an under‐studied micro‐invertebrate group
Source: Ecol Evol. 2020 Dec 9;11(1):309–20. doi: 10.1002/ece3.7042 (PMC7790615; doi:10.1002/ece3.7042)
Supplement: Supplementary file 1 — FigS1‐S18 [file ECE3-11-309-s001.docx]

**Figure S1. Phylogeny comparing mitochondrial assembly methods of historical samples.** Maximum likelihood topology of 109 taxa and 4144 nucleotide and amino acid characters inferred using RAxML (20 heuristic searches and bootstrap of 100 pseudoreplicates). Only Bootstrap values BS >50 are shown. The three different assembly methods are highlighted as follows: NOVOplasty (“Novo” blue font), GetOrganelle (“GO” red font), and SPAdes (“SPAdes” green font). The reference/seed used is in brackets following the assembly method with “SPAdes” indicating that the *de novo* assembly of the same sample was utilized. Note that for several historical samples not all methods were able to provide an assembly of the mitogenome (Table S5). The table to the right shows the individual and total genes recovered (of a maximum of 15) for each sample and method, with a colored box indicating a positive result. The gene order per column is as follows: *atp6, atp8, cox1, cox2, cox3, cytb, nad1, nad2, nad3, nad4, nad4l, nad5, nad6,* rrnL and rrnS. The * to the left and right of a row indicate that the result from this assembly method was utilized in the main phylogeny shown in Fig. 1.

**Figure S2. Phylogeny of *cox1* confirming the placement of *Myriapora truncata* (BLEED1197) from 1871.** Maximum likelihood topology of 68 taxa and 490 amino acid characters inferred using RAxML (20 heuristic searches and bootstrap of 100 pseudoreplicates). Only Bootstrap values BS >50 are shown. The *M. truncata* (BLEED1197) from 1871 and the NCBI sequence (ATX63952) are shown in bold, as is the BS support value for this branch. For % missing characters per taxon see Table S4

**Figure S3. Phylogeny of 18s (nucleotide).** Maximum likelihood topology inferred using RAxML (20 heuristic searches and bootstrap of 100 pseudoreplicates). Only Bootstrap values BS >50 are shown. For % missing characters per taxon see Table S4. *Taxonomical name changes during revision BLEED170 is now *Stephanollona scintillans*, BLEED679 is now *Stephanollona* aff. *scintillans*, and BLEED61 is now *Hincksina* sp. nov.

**Figure S4. Phylogeny of 28s (nucleotide).** Maximum likelihood topology inferred using RAxML (20 heuristic searches and bootstrap of 100 pseudoreplicates). Only Bootstrap values BS >50 are shown. For % missing characters per taxon see Table S4. *Taxonomical name changes during revision BLEED170 is now *Stephanollona scintillans*, BLEED679 is now *Stephanollona* aff. *scintillans*, and BLEED61 is now *Hincksina* sp. nov.

**Figure S5. Phylogeny of atp6 (amino acid).** Maximum likelihood topology inferred using RAxML (20 heuristic searches and bootstrap of 100 pseudoreplicates). Only Bootstrap values BS >50 are shown. For % missing characters per taxon see Table S4. *Taxonomical name changes during revision BLEED170 is now *Stephanollona scintillans*, BLEED679 is now *Stephanollona* aff. *scintillans*, and BLEED61 is now *Hincksina* sp. nov.

**Figure S6. Phylogeny of atp8 (amino acid).** Maximum likelihood topology inferred using RAxML (20 heuristic searches and bootstrap of 100 pseudoreplicates). Only Bootstrap values BS >50 are shown. For % missing characters per taxon see Table S4. *Taxonomical name changes during revision BLEED170 is now *Stephanollona scintillans*, BLEED679 is now *Stephanollona* aff. *scintillans*, and BLEED61 is now *Hincksina* sp. nov.

**Figure S7. Phylogeny of cox2 (amino acid).** Maximum likelihood topology inferred using RAxML (20 heuristic searches and bootstrap of 100 pseudoreplicates). Only Bootstrap values BS >50 are shown. For % missing characters per taxon see Table S4. *Taxonomical name changes during revision BLEED170 is now *Stephanollona scintillans*, BLEED679 is now *Stephanollona* aff. *scintillans*, and BLEED61 is now *Hincksina* sp. nov.

**Figure S8. Phylogeny of cox3 (amino acid).** Maximum likelihood topology inferred using RAxML (20 heuristic searches and bootstrap of 100 pseudoreplicates). Only Bootstrap values BS >50 are shown. For % missing characters per taxon see Table S4. *Taxonomical name changes during revision BLEED170 is now *Stephanollona scintillans*, BLEED679 is now *Stephanollona* aff. *scintillans*, and BLEED61 is now *Hincksina* sp. nov.

**Figure S9. Phylogeny of cob (amino acid).** Maximum likelihood topology inferred using RAxML (20 heuristic searches and bootstrap of 100 pseudoreplicates). Only Bootstrap values BS >50 are shown. For % missing characters per taxon see Table S4. *Taxonomical name changes during revision BLEED170 is now *Stephanollona scintillans*, BLEED679 is now *Stephanollona* aff. *scintillans*, and BLEED61 is now *Hincksina* sp. nov.

**Figure S10. Phylogeny of nad1 (amino acid).** Maximum likelihood topology inferred using RAxML (20 heuristic searches and bootstrap of 100 pseudoreplicates). Only Bootstrap values BS >50 are shown. For % missing characters per taxon see Table S4. *Taxonomical name changes during revision BLEED170 is now *Stephanollona scintillans*, BLEED679 is now *Stephanollona* aff. *scintillans*, and BLEED61 is now *Hincksina* sp. nov.

**Figure S11. Phylogeny of nad2 (amino acid).** Maximum likelihood topology inferred using RAxML (20 heuristic searches and bootstrap of 100 pseudoreplicates). Only Bootstrap values BS >50 are shown. For % missing characters per taxon see Table S4. *Taxonomical name changes during revision BLEED170 is now *Stephanollona scintillans*, BLEED679 is now *Stephanollona* aff. *scintillans*, and BLEED61 is now *Hincksina* sp. nov.

**Figure S12. Phylogeny of nad3 (amino acid).** Maximum likelihood topology inferred using RAxML (20 heuristic searches and bootstrap of 100 pseudoreplicates). Only Bootstrap values BS >50 are shown. For % missing characters per taxon see Table S4. *Taxonomical name changes during revision BLEED170 is now *Stephanollona scintillans*, BLEED679 is now *Stephanollona* aff. *scintillans*, and BLEED61 is now *Hincksina* sp. nov.

**Figure S13. Phylogeny of nad4 (amino acid).** Maximum likelihood topology inferred using RAxML (20 heuristic searches and bootstrap of 100 pseudoreplicates). Only Bootstrap values BS >50 are shown. For % missing characters per taxon see Table S4. *Taxonomical name changes during revision BLEED170 is now *Stephanollona scintillans*, BLEED679 is now *Stephanollona* aff. *scintillans*, and BLEED61 is now *Hincksina* sp. nov.

**Figure S14. Phylogeny of nad4l (amino acid).** Maximum likelihood topology inferred using RAxML (20 heuristic searches and bootstrap of 100 pseudoreplicates). Only Bootstrap values BS >50 are shown. For % missing characters per taxon see Table S4. *Taxonomical name changes during revision BLEED170 is now *Stephanollona scintillans*, BLEED679 is now *Stephanollona* aff. *scintillans*, and BLEED61 is now *Hincksina* sp. nov.

**Figure S15. Phylogeny of nad5 (amino acid).** Maximum likelihood topology inferred using RAxML (20 heuristic searches and bootstrap of 100 pseudoreplicates). Only Bootstrap values BS >50 are shown. For % missing characters per taxon see Table S4. *Taxonomical name changes during revision BLEED170 is now *Stephanollona scintillans*, BLEED679 is now *Stephanollona* aff. *scintillans*, and BLEED61 is now *Hincksina* sp. nov.

**Figure S16. Phylogeny of nad6 (amino acid).** Maximum likelihood topology inferred using RAxML (20 heuristic searches and bootstrap of 100 pseudoreplicates). Only Bootstrap values BS >50 are shown. For % missing characters per taxon see Table S4. *Taxonomical name changes during revision BLEED170 is now *Stephanollona scintillans*, BLEED679 is now *Stephanollona* aff. *scintillans*, and BLEED61 is now *Hincksina* sp. nov.

**Figure S17. Phylogeny of rrnL (nucleotide).** Maximum likelihood topology inferred using RAxML (20 heuristic searches and bootstrap of 100 pseudoreplicates). Only Bootstrap values BS >50 are shown. For % missing characters per taxon see Table S4. *Taxonomical name changes during revision BLEED170 is now *Stephanollona scintillans*, BLEED679 is now *Stephanollona* aff. *scintillans*, and BLEED61 is now *Hincksina* sp. nov.

**Figure S18. Phylogeny of rrnS (nucleotide).** Maximum likelihood topology inferred using RAxML (20 heuristic searches and bootstrap of 100 pseudoreplicates). Only Bootstrap values BS >50 are shown. For % missing characters per taxon see Table S4. *Taxonomical name changes during revision BLEED170 is now *Stephanollona scintillans*, BLEED679 is now *Stephanollona* aff. *scintillans*, and BLEED61 is now *Hincksina* sp. nov.
