# Supplementary material for: A molecular phylogeny of historical and contemporary specimens of an under‐studied micro‐invertebrate group
Source: Ecol Evol. 2020 Dec 9;11(1):309–20. doi: 10.1002/ece3.7042 (PMC7790615; doi:10.1002/ece3.7042)
Supplement: Supplementary file 3 — Supplementary Material [file ECE3-11-309-s003.docx]

***Supporting Information***

A molecular phylogeny of historical and contemporary specimens of an under-studied micro-invertebrate group

***An expanded cheilostome phylogeny***

In presenting our main inferred phylogeny (main text Fig. 1) we only highlight relationships represented by families constituting two or more genera receiving at least moderate support (>70 bootstrap support). The first clade to diverge within the fully supported (100 bootstrap (BS) / 1.00 Posterior Probability (PP)) cheilostomes ingroup (Clade X, Fig. 1) is a highly supported (99 BS /1.00 PP) grouping including *Steginoporella*, *Calpensia*, *Scruparia*, *Membranipora* and *Electra*. The remaining cheilostome taxa form a fully supported group divided into two main sub-clades. The first sub-clade (Clade Y, Fig. 1) is moderately supported (77 BS / 1.00 PP), and further split into two groups. The basal of these two groupings is a fully supported lineage of two bugulids. The candids comprising *Cradoscrupocellaria* and *Emma* have moderate support (88 BS / 1.00 PP), but place paraphyletic to a highly supported (95 BS / 1.00 PP) monophyly of two *Caberea* species, from the same family. The terminal of these two groupings receives moderate support (84 BS / 1.00 PP) and in ascending order, we recover a lineage of three hippothoids with high support (90 BS / 1.00 PP), the adeonids represented by five genera with full support, and a flustrid clade, again fully supported and harboring three genera. The second main sub-clade (Clade Z, Fig. 1) is highly supported (98 BS / 1.00 PP), and further splits into multiple lineages. Again, in ascending order, a fully supported lineage of three catenicellid genera that form a highly supported (99 BS / 1.00 PP) sister relationship with *Myriapora truncata*. A moderately supported (78 BS / 0.99 PP) grouping of *Fenestrulina*, *Microporella* and *Tessaradoma* (98 BS / 1.00 PP). A highly supported (96 BS/ 1.00 PP) monophyly of four celleporids. A fully supported philodoporid grouping comprises two genera and four species. A monophyly comprising a fully supported grouping of three *Parasmittina* species is observed. And finally, a highly supported (97 BS / 1.00 PP) clade formed by the species *Oshurkovia littoralis* with members of *Porella*.

In general, our tree (Fig. 1) lends statistical support to previous work on the molecular phylogeny of cheilostomes (Knight, Gordon, & Lavery, 2011; Orr, Haugen, et al., 2019; Orr, Waeschenbach, et al., 2019; Waeschenbach, Taylor, & Littlewood, 2012), but also demonstrates well-supported branching patterns for new relationships. In summary, the most basal cheilostome clade (Clade X, Fig. 1) consists of *Electra*, *Membranipora* and *Scruparia*, reminiscent of the topology depicted by Waeschenbach et al. (2012), but we add two newly sequenced genera, namely, *Calpensia* and *Steginoporella* to this grouping. *Calpensia* (currently placed in Microporidae, but also historically assigned to Calpensiidae, Canu & Bassler, 1923) is sister to *Steginoporella,* based on 14 and 16 genes respectively (Fig 1)*.* They share a frontal, depressed, pseudoporous cryptocyst, which in *Calpensia* and *Thalamoporella*, the sister of *Steginoporella* (Knight et al., 2011), bears two opesiules while in *Steginoporella* are paired opercular indentations, outlined by the median process, forming opesiule-like lateral openings in some species (see SEMs in SI). This result calls for a revision of the systematic placement of *Calpensia*/Calpensiidae, likely in the superfamily Thalamoporelloidea, where additional molecular sequence data from *Thalamoporella* could prove useful*.* Presently, only partial *cox1* and rrnL sequence data are available each from two different *Thalamoporella* species, and as such, were not included in this study. In the first main cheilostome clade (Clade Y, Fig. 1) we add the genera *Arachnopusia, Bicellariella, Caberea, Emma, Hincksina, Hippothoa*, *Patsyella* and *Rhabdozoum* to that previously shown (Orr, Haugen, et al., 2019; Waeschenbach et al., 2012). *Hippothoa*, a morphologically highly distinct hippothoid, places with high confidence with the previously sequenced *Antarctothoa* and *Celleporella* (Knight et al., 2011). *Hincksina* places with commonly occurring members of the family Flustridae (namely *Flustra* and *Securiflustra*). Even though *Hincksina* is in need of revision with regards to its relationship with *Gregarinidra,* the placement of this sample within the Flustridae seems beyond doubt. Moving on to the second main clade (Clade Z, Fig. 1) of cheilostomes represented in our topology, we place *Cornuticella, Myriapora, Omalosecosa, Parasmittina, Porella, Pterocella, Stephanollona, Terminocella, Tessaradoma and Turbicellepora* for the first time. *Myriapora* places as sister to the catenicellids, a position that may be the result of a limited taxon sample. Catenicellidae have a fundamentally gymnocystal-shield, with the earliest, Maastrichtian, species having a costate frontal area (Banta & Wass, 1979). In contrast, Myriaporidae have a pseudoporous-lepralioid-shield (see SEMs in SI) and, in addition, a vastly different astogeny and colony form is observed between these two families (Ferretti, Magnino, & Balduzzi, 2007; Wass, 1983). *Tessaradoma boreale* (family Tessaradomidae) places between *Microporella* and *Fenestrulina*, two genera that are superficially similar but are now found to belong to separate families (Orr, Waeschenbach, et al., 2019). *Tessaradoma*, prior to the introduction of the family Tessaradomidae (also including the genus *Smithsonius*), has been considered within Microporellidae (MacGillivary, 1895) despite these families having contrasting frontal-shield ontogenies. A key feature of *Tessaradoma*, which partly explains its attributions to Microporellidae (among other families), is a suboral opening in the frontal shield interpreted as a spiramen (Gordon, 1993). However, broader taxon sampling is needed to confirm the affinity of *Tessaradoma* to Microporellidae, prompting a future inclusion of Taylorus, as previously suggested (Orr, Waeschenbach, et al., 2019), *Smithsonius,* and lastly *Siphonicytara*, which may have descended from a *Beisselina*-like (Tessaradomidae) ancestor (Gordon & Taylor, 2015). *Turbicellepora* (Ryland, 1963)*,* unlike *Omalosecosa* (Canu & Bassler, 1925), shows presence of an orbicular orifice with a sinus in addition to a perforate ooecium. Both taxa, when first introduced, were placed as *Cellepora*, the type genus of their designated family (Celleporidae), and later assigned to separate genera within the same family, based on the morphological characters emphasized. This position may have nomenclatural implications if, upon improved taxon sampling, *Turbicellepora* and *Omalosecosa* are shown to be species within the same genus. In this case, *Turbicellepora* would be subsumed in *Omalosecosa* (the earlier named genus).

References cited

Banta, W. C., & Wass, R. E. (1979). Catenicellid cheilostome Bryozoa. I. Frontal walls. *Australian Journal of Zoology Supplementary Series, 27*(68), 1-70. doi:10.1071/AJZS068

Canu, F., & Bassler, R. S. (1923). North American later Tertiary and Quaternary Bryozoa. *United States National Museum Bulletin, 125*, 1-302.

Canu, F., & Bassler, R. S. (1925). Les Bryozoaires du Maroc et de Mauritanie. *Mémoires de la Société des Sciences Naturelles du Maroc, 10*.

Ferretti, C., Magnino, G., & Balduzzi, A. (2007). Morphology of the larva and ancestrula of *Myriapora truncata* (Bryozoa, Cheilostomatida). *Italian Journal of Zoology, 74*(4), 341-350. doi:10.1080/11250000701629572

Gordon, D. P. (1993). Bryozoan frontal shields: studies on umbonulomorphs and impacts on classification. *Zoologica Scripta, 22*(2), 203-221. doi:10.1111/j.1463-6409.1993.tb00352.x

Gordon, D. P., & Taylor, P. D. (2015). Bryozoa of the Early Eocene Tumaio Limestone, Chatham Island, New Zealand. *Journal of Systematic Palaeontology, 13*(12), 983-1070. doi:10.1080/14772019.2014.991905

Knight, S., Gordon, D. P., & Lavery, S. D. (2011). A multi-locus analysis of phylogenetic relationships within cheilostome bryozoans supports multiple origins of ascophoran frontal shields. *Molecular Phylogenetics and Evolution, 61*(2), 351-362. doi:<http://dx.doi.org/10.1016/j.ympev.2011.07.005>

MacGillivary, P. H. (1895). A monograph of the Tertiary Polyzoa of Victoria. In *Transactions of the Royal Society of Victoria* (Vol. 4, pp. 1-166).

Orr, R. J. S., Haugen, M. N., Berning, B., Bock, P., Cumming, R. L., Florence, W. K., . . . Liow, L. H. (2019). A genome-skimmed phylogeny of a widespread bryozoan family, Adeonidae. *BMC Evolutionary Biology, 19*(1), 235. doi:10.1186/s12862-019-1563-4

Orr, R. J. S., Waeschenbach, A., Enevoldsen, E. L. G., Boeve, J. P., Haugen, M. N., Voje, K. L., . . . Liow, L. H. (2019). Bryozoan genera *Fenestrulina* and *Microporella* no longer confamilial; multi-gene phylogeny supports separation. *Zoological Journal of the Linnean Society, 186*(1), 190-199. doi:10.1093/zoolinnean/zly055

Ryland, J. S. (1963). Systematic and biological studies on Polyzoa (Bryozoa) from western Norway. *Sarsia, 14*(1), 1-59. doi:10.1080/00364827.1963.10409518

Waeschenbach, A., Taylor, P. D., & Littlewood, D. T. J. (2012). A molecular phylogeny of bryozoans. *Molecular Phylogenetics and Evolution, 62*, 718-735.

Wass, R. E. (1983). Early astogeny in the Catenicellidae (Bryozoa, Cheilostomata). *Alcheringa: An Australasian Journal of Palaeontology, 7*(1), 41-48. doi:10.1080/03115518308619632
